# A Case of Mumps Presenting With Unilateral Submandibular Sialadenitis and Laryngeal Edema

**DOI:** 10.7759/cureus.29290

**Published:** 2022-09-18

**Authors:** Go Ogawa, Ryoji Kagoya, Masato Mochiki, Ken Ito

**Affiliations:** 1 Otolaryngology, Teikyo University, Tokyo, JPN; 2 Otorhinolaryngology-Head and Neck Surgery, The University of Tokyo, Tokyo, JPN

**Keywords:** cervical cellulitis, submandibular gland, unilateral, laryngeal edema, mumps

## Abstract

Mumps is a viral infection that primarily affects the parotid glands. Here, we report an atypical case of mumps presenting with unilateral submandibular sialadenitis and laryngeal edema. A 20-year-old woman with unremarkable medical history was referred to our hospital for the management of left submandibular sialadenitis. Laryngeal endoscopy revealed laryngeal edema. Contrast-enhanced computed tomography of the neck revealed swelling of the left submandibular gland with surrounding fluid density and increased density of the cervical subcutaneous adipose tissue. A few days later, both anti-mumps immunoglobulin M (IgM) and IgG antibodies were positive, and she was diagnosed with mumps. To date, there have been no reports of unilateral submandibular gland mumps complicated by laryngeal edema. It is important to keep in mind that the involvement of the submandibular gland in cases of mumps is probably a risk factor for laryngeal edema.

## Introduction

Mumps is a common viral infection that presents with painful swelling of salivary glands. The most common symptom is swelling of the parotid glands, which is observed in 95% of the symptomatic cases [1]; approximately 90% of parotid swellings are bilateral [1]. Additionally, combined swelling of the parotid and submandibular glands is observed in 11% of the cases [2]. Isolated involvement of the submandibular glands is rare [3,4]. Although mumps is generally a mild disease, it can cause various complications, including encephalitis, meningitis, orchitis, myocarditis, pancreatitis, nephritis, and unilateral deafness [5]. Laryngeal edema is a rare complication of mumps, and only a few cases of it accompanying mumps have been reported [2,6-8]. Here, we report a rare case of unilateral submandibular gland mumps accompanied by edematous changes in the larynx and cervical cellulitis.

## Case presentation

A 20-year-old woman with an unremarkable medical history presented to a primary care clinic with a sore throat and pain on the left side of the neck. She was diagnosed with acute pharyngitis and received antimicrobial treatment for three days. As her symptoms did not improve, she visited another clinic and was diagnosed with left submandibular sialadenitis based on the swelling of the left lower jaw. She was referred to our hospital and admitted for further treatment. Although she had difficulty swallowing due to sore throat, she had no hoarseness and airway obstructive symptoms including dyspnea and wheezing. She denied any history of mumps, vaccination, or submandibular gland sialolithiasis. The swollen area extended from the left submandibular region to the supraclavicular region, along the anterior neck. Intraoral inspection revealed a purulent discharge from the opening of the left Wharton’s duct. Based on these findings, bacterial submandibular sialadenitis was considered.

**Figure 1:**
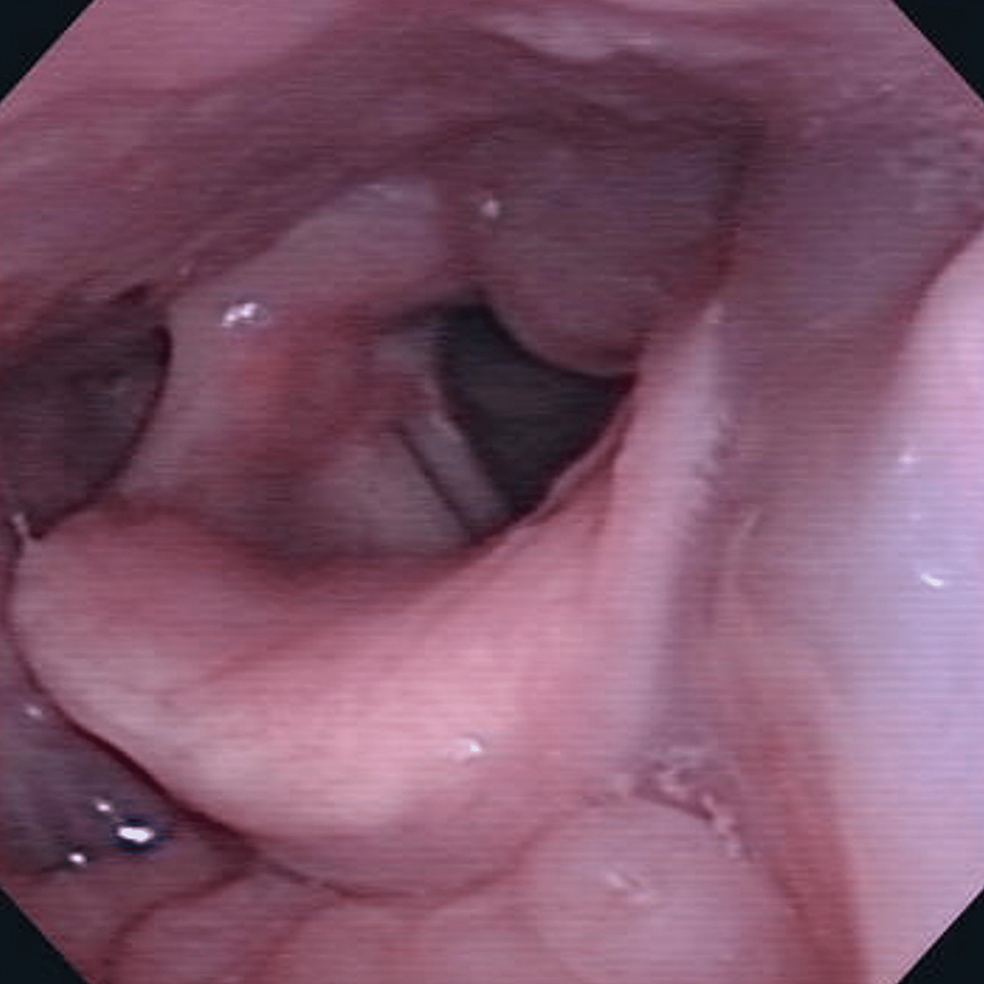
Endoscopic findings of the larynx at the first visit Edematous swelling of the left lateral wall of the hypopharynx, epiglottis, and left pyriform sinus are observed.

**Figure 2:**
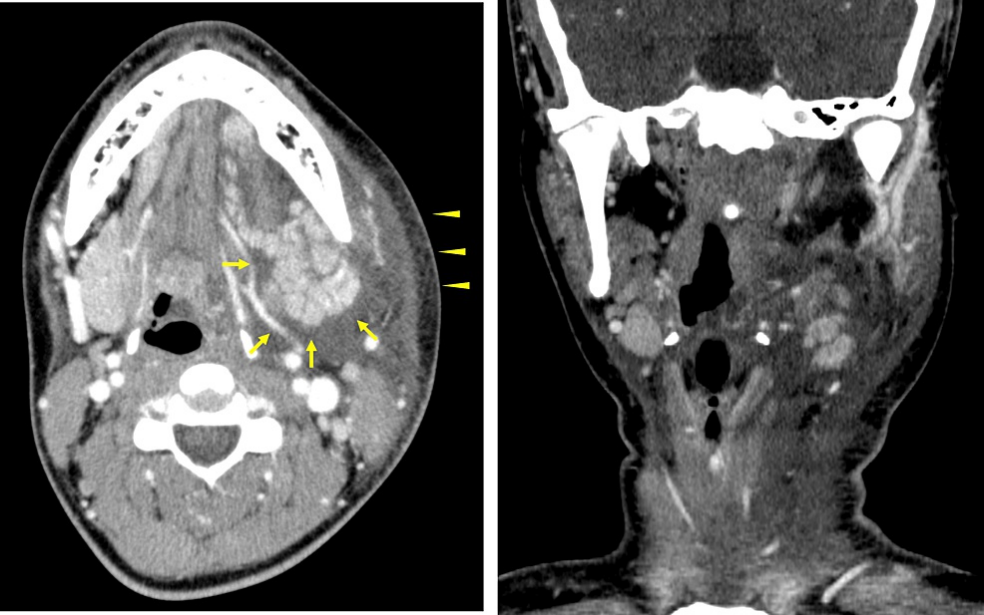
Contrast-enhanced neck CT images (axial and coronal views) Swelling and lobulation of the left submandibular gland with surrounding fluid density (arrow) and increased density of the cervical subcutaneous adipose tissue (arrowhead) are observed. CT: computed tomography

Laryngeal endoscopy revealed edematous swelling of the left lateral wall of the hypopharynx, epiglottis, and left pyriform sinus (Figure 1). A contrast-enhanced computed tomography (CT) of the neck revealed swelling and lobulation of the left submandibular gland with surrounding fluid density and increased density of cervical subcutaneous adipose tissue (Figure 2). Parotid gland findings were normal. Laboratory test results showed normal white blood cell count (WBC) with mild neutrophilia, normal level of serum amylase, and a slightly elevated level of C-reactive protein (CRP) (Table 1). Although a viral infection was suspected on the basis of the blood test results, bacterial submandibular sialadenitis and subsequent neck cellulitis could also be suggested, considering the findings of CT. Therefore, the patient was treated with doripenem (3.0 g) and hydrocortisone (300 mg) for three days. Following the initiation of treatment, the laryngeal edema and neck swelling gradually improved. On the fourth day of hospitalization, both anti-mumps immunoglobulin M (IgM) and IgG antibodies were positive (Table 1), and she was diagnosed with mumps. Therefore, antibacterial treatment was stopped at the time of diagnosis. Bilateral preauricular swelling appeared only for one day, on the third day of hospitalization, probably due to the parotid gland involvement of mumps. The patient was discharged on the seventh day of hospitalization.

**Table 1:** Result of blood tests WBC: white blood cell; CRP: C-reactive protein; Ig: immunoglobulin

Item	Result	Reference range
WBC (/μl)	4600	3300–8600
Neutrophil (%)	76	54–72
Lymphocyte (%)	13	21–35
Monocyte (%)	10	4–8
Amylase (IU/L)	124	44–132
CRP (mg/dL)	0.06	0.00–0.14
Anti-mumps IgM	+	－
Anti-mumps IgG	+	－

## Discussion

Mumps is an infectious disease of the salivary glands seen in children. It is caused by the mumps virus, which can also infect adults. It is commonly encountered in otolaryngology, pediatrics, and internal medicine.

Laryngeal edema associated with mumps is relatively rare. There are only a few reports of this condition [2,6-8]. Table 2 summarizes the reported cases and our case. In all reported cases, apparent swelling of both the parotid and submandibular glands was observed at the onset [2,6-8]. In contrast, our case presented with inflammation of only one submandibular gland, however, late-onset swelling of the bilateral parotid glands was observed for only one day. To our knowledge, there are no reported cases of unilateral submandibular gland mumps accompanied by laryngeal edema. Although submandibular gland swelling is observed in approximately 10% of patients with common mumps [2,8], it has been confirmed in all the reported cases of mumps associated with laryngeal edema. Therefore, swelling of the submandibular gland may be a risk factor for laryngeal edema. If submandibular gland swelling is observed, the possibility of laryngeal edema should be considered.

**Table 2:** Summary of the reported cases of mumps with laryngeal edema PG: parotid gland; SMG: submandibular gland; WBC: white blood cell; CRP: C-reactive protein; Amy: amylase

	Age	Sex	PG swelling	SMG swelling	WBC (/μL)	CRP (mg/dL)	Amy (IU/L)	Tracheostomy
Ishida, et al. [2]	43	F	left	bilateral	7800	0.4	452	－
Ishida, et al. [2]	36	F	bilateral	bilateral	4300	0.5	119	+
Ishida, et al. [2]	23	F	bilateral	bilateral	3360	0.2	668	+
Iizuka, et al. [6]	27	M	bilateral	bilateral	7200	1.44	1250	－
Nakao, et al. [7]	45	M	bilateral	bilateral	no data	no data	no data	－
Ohki, et al. [8]	31	F	bilateral	bilateral	4100	0.2	no data	－
Ohki, et al. [8]	16	M	bilateral	bilateral	3500	1.5	no data	－
Ohki, et al. [8]	31	F	bilateral	bilateral	3900	2.6	no data	－
Ohki, et al. [8]	40	F	left	bilateral	6800	1.1	no data	－
Ohki, et al. [8]	25	M	bilateral	bilateral	5900	1.0	no data	－
Ogawa, et al.	20	F	none	left	4600	0.06	124	－

Blood test results showed a normal WBC count and minor elevation of CRP levels in all the cases, including ours, despite airway obstruction. In this case, swelling of the submandibular gland with surrounding fluid density and increased density of the cervical subcutaneous adipose tissue were also confirmed by CT of the neck. In cases of mumps with submandibular gland swelling, edematous changes due to blood stasis may be likely to occur despite mild inflammation.

## Conclusions

We report a rare case of unilateral submandibular gland mumps associated with laryngeal edema and cervical cellulitis. Submandibular gland swelling may be a risk factor for laryngeal edema. This is the first case report of unilateral submandibular gland mumps associated with laryngeal edema. The present case adds to the current knowledge by emphasizing that unilateral submandibular gland mumps can cause laryngeal edema.
